# 3′UTRs Regulate Mouse *Ntrk2* mRNA Distribution in Cortical Neurons

**DOI:** 10.1007/s12031-020-01579-8

**Published:** 2020-05-19

**Authors:** Shangqin Chen, Jinjin Zhu, Peijun Li, Zhaonan Xia, Mengjing Tu, Zhenlang Lin, Baoji Xu, Xiaoqin Fu

**Affiliations:** 1 The Second Affiliated Hospital and Yuying Children’s Hospital, Wenzhou Medical University, Wenzhou, 325000 Zhejiang China; 2 Department of Neuroscience, The Scripps Research Institute, 130 Scripps Way, Jupiter, FL 33458 USA

**Keywords:** *NTRK2*, 3′UTR, mRNA, Neuron

## Abstract

There are two major isoforms of NTRK2 (neurotrophic receptor tyrosine kinase 2, or TrkB), full-length isoform with tyrosine kinase (TK) domain intact (+) and spliced isoform without tyrosine kinase domain (TK(−)). Within each isoform, there exist subtypes with minor modifications of the protein sequences. In human, the *NTRK2* mRNA transcripts encoding TK(+) have same 3′UTRs, while the transcripts encoding subtypes of NTRK2 TK(−) have two completely different 3′UTRs. In mouse, the mRNA transcripts encoding same NTRK2 protein sequence for either TK(+) or TK(−) have long or short 3′UTRs, respectively. The physiological functions of these different 3′UTRs are still unknown. Pilocarpine stimulation increased *Ntrk2* mRNA levels in soma, while the increase in synaptosome was smaller. FISH results further showed that mouse *Ntrk2* transcripts with different 3′UTRs were distributed differently in cultured cortical neurons. The transcripts with long 3′UTR were distributed more in apical dendrites compared with transcripts with short 3′UTR. Our results provide evidence of non-coding 3′UTR function in regulating mRNA distribution in neurons.

**Electronic supplementary material:**

The online version of this article (10.1007/s12031-020-01579-8) contains supplementary material, which is available to authorized users.

## Introduction

With the development of genomic sequencing technology, it is well-known that the majority of transcribed mammalian genomes do not encode proteins (Alexander et al. 2010). Many of those non-coding transcripts are called non-coding RNAs (ncRNAs). In addition, transcripts encoding proteins contain non-coding RNA sequences at both 5′ and 3′ ends, which are called 5′ untranslated regions (5′UTR) and 3′ untranslated regions (3′UTR), respectively. Over the past two decades, our understanding of ncRNAs or non-coding RNA sequences has changed from no function to diverse functions (Bartoszewski and Sikorski 2018; Geissler and Grimson 2016; Hogg and Harries 2014; Roberts et al. 2014; Zampetaki et al. 2018). 5′UTR or 3′UTR sometimes coordinates with ncRNAs to fulfill a common function or spatiotemporally regulate gene expression (Bartoszewski and Sikorski 2018; Jia et al. 2013; Leppek et al. 2018; Oliva et al. 2015). For example, miRNAs, a class of short ncRNAs (22–25 nt), can recognize specific target mRNA sequences frequently localized within the 3′UTR and initiate translational repression (Chekulaeva and Filipowicz 2009; Djuranovic et al. 2012). Some ncRNAs are directly processed from 3′UTR (Chao and Vogel 2016; Kawano et al. 2005; Zhao et al. 2018).

Studies indicate that many genes undergo alternative cleavage and polyadenylation to generate transcripts with identical open reading frames but distinct 3′UTRs (Andreassi et al. 2018; Zhang et al. 2005). Such phenomenon is particularly widespread in the central nervous system of mammals (Kocabas et al. 2015; Miura et al. 2013; Oktaba et al. 2015). Neuron is morphologically complex and relies heavily on its extraordinary cytoarchitectural structure including axons and dendrites to accomplish its functions. To maintain its morphological structure, neurons depend on different transports and expressions of proteins at different subcellular compartments. Portion of this task is achieved by local translation (Holt and Schuman 2013). Increasing evidences demonstrate that some mRNAs, with the help of their 3′UTRs, are not directly translated at soma but transported to dendrites where these mRNAs are translated to proteins in response to different activities (An et al. 2008; Bramham and Wells 2007; Kobayashi et al. 2005; Middleton et al. 2019; Rook et al. 2000; Taliaferro et al. 2016; Tongiorgi et al. 2004; Tushev et al. 2018). Despite these findings, our understanding of the mechanism is far from complete.

Brain-derived neurotrophic factor (BDNF) is one of the most extensively studied neurotrophins in the mammalian brain. It is involved in a wide range of neurophysiological processes, including neuroprotection, development of neurons and glial cells, and modulation of synaptic interactions (Foltran and Diaz 2016; Kang et al. 1997; Numakawa et al. 2010). The effects of BDNF are regulated by its spatiotemporal expression and association with its high affinity receptor NTRK2 (neurotrophic receptor tyrosine kinase 2, or TrkB) (Binder and Scharfman 2004; Fenner 2012; Kang et al. 1997; Klein et al. 1991; Yamada and Nabeshima 2003). *NTRK2* gene encodes two major isoforms, a full-length receptor and a truncated receptor, which does not have the intracellular tyrosine kinase domain (TK(−)) (Klein et al. 1990; Stoilov et al. 2002). Activated full-length NTRK2 triggers its intrinsic tyrosine kinase activity, which leads to the activation of downstream intracellular signaling cascades to induce differentiation, proliferation, and survival (Reichardt 2006). The NTRK2 TK(−) is also abundantly expressed in the brain (Klein et al. 1990), and was originally thought that NTRK2 (−) mainly inhibits full-length NTRK2 signaling. However, NTRK2 TK(−) has additional functions to translocate BDNF, induces neurite outgrowth, and modifies cytoskeletal structures (Fenner 2012). Various neurological diseases are associated with malfunction of NTRK2, including neurological diseases, cancers, obesity, and eating disorders (Altar et al. 2009; Desmet and Peeper 2006; Farooqi and O’Rahilly 2006).

Mouse *Ntrk2* mRNAs encoding for both full-length and TK(−) isoforms have short or long 3′UTRs, and were detected in dendrites of cultured cortical neurons (Tongiorgi et al. 2004). It is not known, however, whether the 3′UTRs play any roles in dendritic location of *Ntrk2* mRNAs. In this study, we compared the differences between human and mouse *NTRK2* transcripts and examined the expression levels of different mouse *Ntrk2* mRNA variants in soma and synaptosome of mouse cortex with or without pilocarpine treatment. We also evaluated the effects of 3′UTRs on the subcellular distribution of mouse *Ntrk2* transcripts in cultured cortical neurons. We conclude that *Ntrk2* 3′UTRs influence subcellular distribution of associated transcripts in neurons.

## Materials and Methods

### Animals and Pilocarpine Treatment

All animal care and experiments were conducted in strict accordance with the Guidelines approved by the Animal Experimental Ethical Inspection of Laboratory Animal Centre of Wenzhou Medical University (approval number wydw2019-0723). Mouse strains (C57BL6/J) were originally purchased from Beijing Vital River Laboratory Animal Technology Co., Ltd. and housed under specific pathogen-free conditions at the laboratory animal center of Wenzhou Medical University. For pilocarpine treatment, 2-month old male mice were anesthetized with urethane (0.5 g/kg, Cat#A600448-0250, Shenggong Biotech Co. Ltd., Shanghai, China) 30 min prior to intraperitoneal injection of 300 mg/kg pilocarpine (Cat#HY-B0726, MedChemExpress, Monmouth Junction, NJ, USA) or saline. Animals were sacrificed 3 h later.

### Generation of GFP-Mouse *Ntrk2* 3′UTR Constructs

The genomic sequences encoding mouse *Ntrk2* 3′UTRs-A, B, and AB were obtained by PCR using mouse genomic DNA as templates. The sequences of the primers used for the three constructs were shown in Table 3. The forward and reverse primers contain BstXI and KpnI restriction sites, respectively. PCR products for the three *Ntrk2* 3′UTRs were digested with BstXI and KpnI restriction enzymes and subcloned into the pcDNA3.1(−) (Invitrogen, Carlsbad, CA) plasmid containing EGFP coding sequence at the same sites to generate GFP-A, GFP-B, and GFP-AB constructs. All plasmids were sequenced to make sure that no mutations were introduced by PCR.

### Primary Cell Culture and Transfection

Cortical neurons were isolated and cultured according to a previously described procedure (Sala et al. 2000) from E18.5 mouse embryos. We used lipofectamine 2000 to transfect cultured neurons according to manufacturer’s instructions.

### In Situ Hybridization

In situ hybridization of cultured neurons was performed with DIG-labeled riboprobes and the TSA Plus Fluorescein System (PerkinElmer, Waltham, MA) according to previously described procedures (An et al. 2008; Marz et al. 1998; Muddashetty et al. 2007) with modifications. To generate antisense and sense riboprobes, four mouse cDNA sequences located in mouse *Ntrk2* 3′UTR-A (nucleotides 2926–4603 of NM_001025074.1), 3′UTR-AB (4720-8711 of and NM_001282961.1), 3′UTR-C (nucleotides 1432-2484 of M33385), and 3′UTR-CD (nucleotides 3112-7020 of NM_008745.3) were amplified by PCR, cloned into the pBluscript II KS(−) plasmid (Stratagene, Cedar Creek, TX, USA) and used as template for FISH probe 1, 2, 3, and 4, respectively. Antisense and sense RNA probes were synthesized from linearized plasmids by using T3 and T7 RNA polymerases, respectively. Probes recognizing BGH mRNA or BDNF mRNA with long 3′UTR were used as controls to show somatic or dendritic distributions of mRNA, respectively (An et al. 2008). After synthesis, riboprobes were fragmented to ~ 0.1 kb by alkaline hydrolysis, and used for FISH analyses. Cells were fixed for 30 min in 4% paraformaldehyde, washed in PBST, permeabilized with 0.2% Triton X-100 in PBS for 5 min, and acetylated for 10 min with 0.1 M triethanolamine hydrochloride/0.25% acetic anhydride (pH 8.0). Probe concentrations were 500 ng/ml for *Ntrk2* mRNA and 100 ng/ml for GFP mRNA. After overnight hybridization, cells were treated with 25 μg/ml RNase A at 37 °C for 20 min and washed twice at 65 °C in 0.1X SSC for 30 min. Fluorescent MAP2 immunocytochemistry was performed after the hybridization step was completed. For all in situ hybridization results, images from an antisense probe and its sense control probe were taken using confocal microscope with the same settings. The settings were adjusted to avoid the saturation of signals especially in soma. The sense in situ hybridization signal was considered the background and used as normalization for the antisense signal. The soma was outlined, and a series of equal rectangles were drawn along dendrites to measure the average intensity of in situ hybridization signals by using the NIH image software (ImageJ). After the hybridization and wash steps, green fluorescence from GFP in transfected neurons was not detectable.

### Synaptosome Preparation from Mouse Cortex

Two-month old male mice cortex were rapidly removed from brain and placed in 10 volumes of ice-cold buffer containing 10 mM Tris-HCl (pH 7.4), 2 mM EGTA, and 320 mM sucrose with protease inhibitors. The tissue was homogenized in a Teflon/glass homogenizer (clearance 0.1–0.15 mm) by eight gentle up and down strokes at 800 rpm. The homogenate was spun at 4 °C, 1500 *g* for 10 min. The pellet (P1) contains mainly cell bodies. The supernatant was recovered and centrifuged again at 4 °C, 28,000×*g* for 15 min to produce a pellet (P2) containing synaptosome. Total RNA was immediately extracted from both P1 and P2 using Trizol (Life Technologies, Carlsbad, CA, USA).

### Quantitative Real-time PCR

Quantitative real-time PCR was performed using ReverTra Ace qPCR RT Kit (Cat#FSQ-101) according to manufacturer’s instruction (Toyobo Co. Ltd., OSAKA, Japan) in a CFX96 Real-time System (Bio-rad) with the following program: 95 °C for 3 min; 95 °C 15 s, 60 °C 30 s for 40 cycles; 65 to 95 °C (gradient of 0.5 °C) (melting curve step). Delta Ct values were obtained using β-actin as reference gene. The sequences of primers used to detect different mRNAs are shown in Table 3.

### Statistical Analysis

All data were expressed as mean ± SEM of at least three independent experiments. Statistical analyses were performed using GraphPad Prism 7.0 (GraphPad Software Inc., San Diego, CA, USA). Statistical significance was determined by one-way analysis of variance (ANOVA) followed by Tukey’s test when analyzing more than two groups. Student’s *t* test was used for the comparisons of two groups. *p* < 0.05 was considered significant (*means *p* < 0.05; #means *p* < 0.01).

## Results

### Comparison of *NTRK2* mRNA between Human and Mouse

**Fig. 1:**
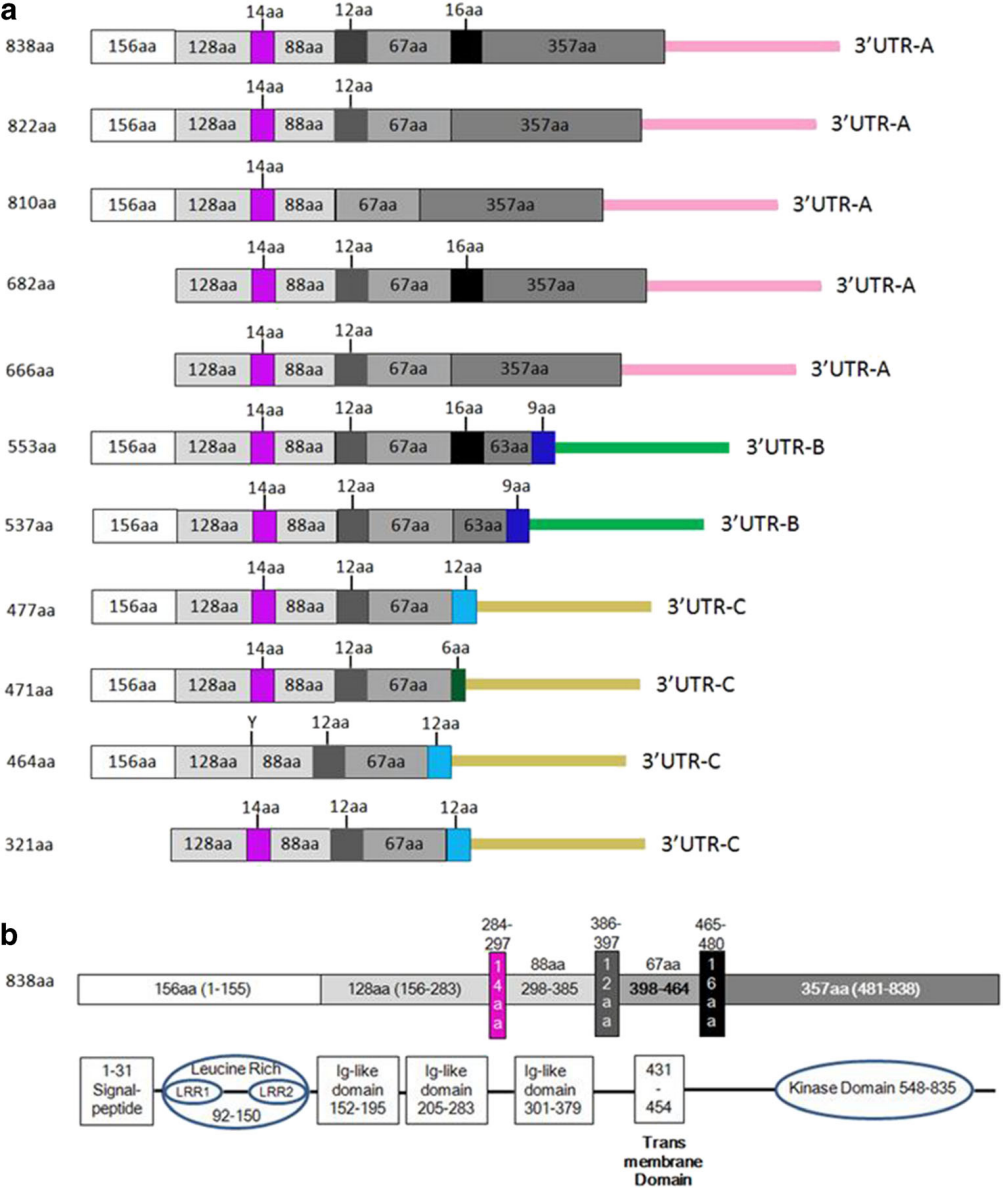
Diagram of human *NTRK2* mRNA variants and protein structure. **a** Diagram of human *NTRK2* mRNA variants, which encode 11 isoforms of NTRK2 proteins. Translated regions are shown by boxes, and 3′ untranslated regions are shown by colored lines. Box with the same color or shape has the same mRNA sequence and amino acid number for each segment is shown. Colored line representing 3′UTR with the same color has same mRNA sequence. These 11 variants have three different kinds of 3′UTR. **b** Schematic structure of 838aa human NTRK2 protein

**Fig. 2:**
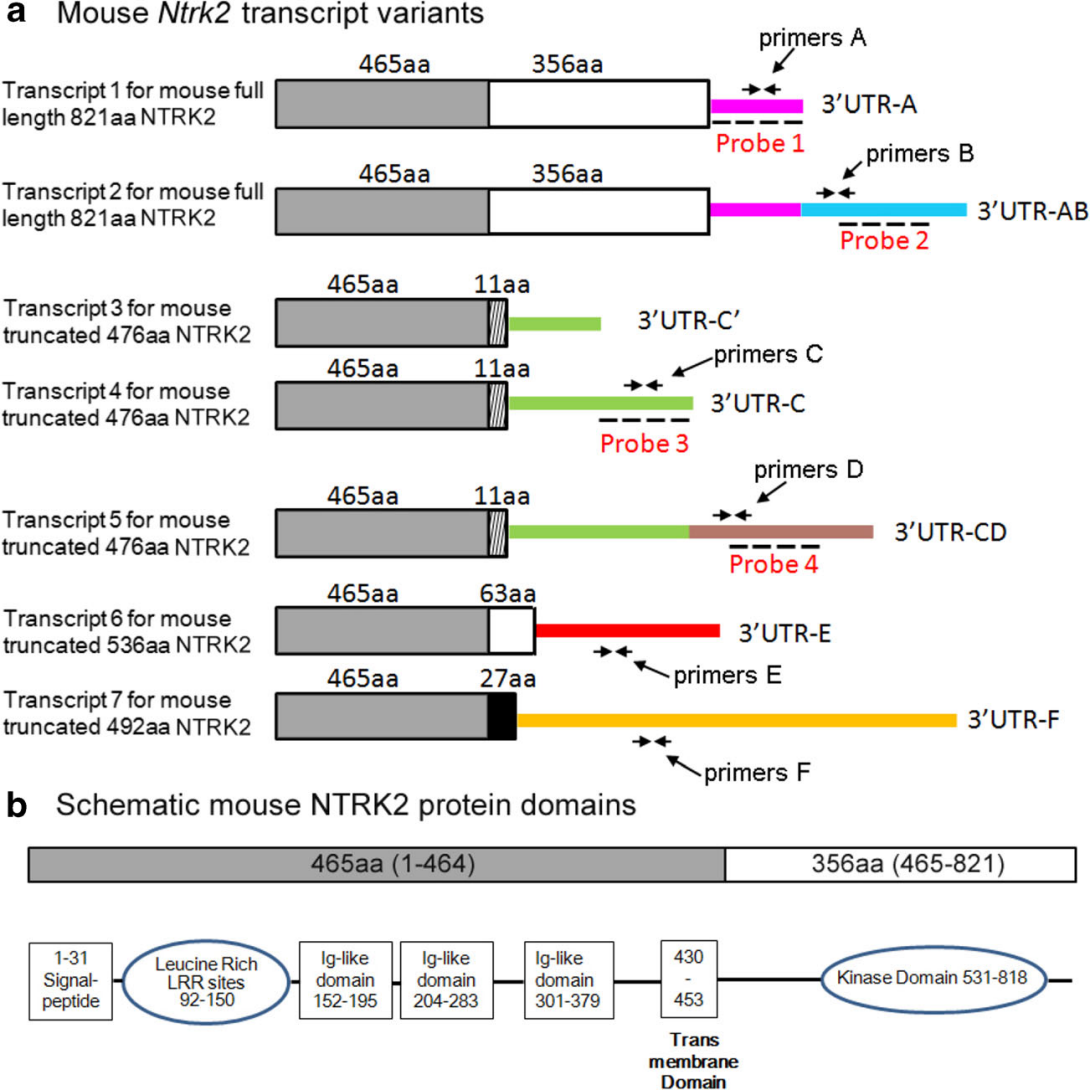
Diagram of mouse *Ntrk2* mRNA variants and protein structure. **a** Diagram of seven mouse *Ntrk2* mRNA variants which encode four isoforms of mouse NTRK2 proteins (821, 476, 536, 492 aa). Translated regions are shown by boxes, and 3′ untranslated regions are shown by colored lines. Box with the same color or shape has the same mRNA sequence. Amino acid number of coded protein segment is shown on top of each box. Colored line with the same color has the same RNA sequence. These seven variants have seven different kinds of 3′UTR. The locations of probes used in FISH experiment are shown in the figure. Probe 1 detects transcripts with 3′UTR-A or 3′UTR-AB. Probe 2 only detects transcript with 3′UTR-AB. Probe 3 detects transcripts with both 3′UTR-C and 3′UTR-CD. Probe 4 only detects transcripts with 3′UTR-CD. Primers used in QPCR are also shown in the picture. **b** Schematic structure of 821aa mouse NTRK2 protein domains

From NCBI database, we found 11 human NTRK2 isoforms with different molecular weights, which are translated from at least 11 mRNA variants (Table 1 and Fig. 1). Human NTRK2 isoforms include five NTRK2 isoforms containing tyrosine kinase domains (832aa, 822aa, 810aa, 682aa, and 666aa) and six NTRK2 isoforms without tyrosine kinase domain (553aa, 537aa, 477aa, 471aa, 464aa, and 321aa). The mRNA variants encoding NTRK2 isoforms with tyrosine kinase domain have same 3′UTR (3′UTR-A). The mRNA variants encoding human NTRK2 isoforms without tyrosine kinase domain have two different 3′UTR: 3′UTR-B and 3′UTR-C (Table 1 and Fig. 1). In mouse, there are four NTRK2 isoforms with different molecular weights, which are translated from at least seven mRNA variants (Table 2 and Fig. 2). Mouse NTRK2 isoforms include one full-length NTRK2 isoform containing tyrosine kinase domain and three truncated NTRK2 isoforms without tyrosine kinase domain (476aa, 536aa, and 492aa). The mRNA variants encoding mouse full-length NTRK2 have two kinds of 3′UTRs (3′UTR-A and 3′UTR-AB). The mRNA variants encoding mouse truncated 476aa NTRK2 isoform have three kinds of 3′UTRs (3′UTR-C′, 3′UTR-C, and 3′UTR-CD). The other two mRNA variants encoding mouse truncated 536aa or 492aa NTRK2 isoforms have 3′UTR-E or 3′UTR-F, respectively. If including different 5′UTRs, there will be more mRNA variants encoding NTRK2 in both human and mouse. Despite the differences between human *NTRK2* and mouse *Ntrk2* mRNAs, the protein structure of full-length NTRK2 for either human or mouse is very similar (Figs. 1b and 2b). The protein sequence of 821aa mouse NTRK2 isoform is 93% similar to those of 822aa human NTRK2 isoform (data not shown).

**Table 1:** Human *NTRK2* mRNA variants

Human *NTRK2* mRNA variants			
ID	Tyrosine kinase (TK) +/–	Coded protein	3′UTR (bp)
NM_006180.4	TK+	838aa	A (5617)
NM_001018064	TK+	822aa	A (5617)
NM_001369534	TK+	810aa	A (5617)
NM_001369536	TK+	682aa	A (5617)
NM_001369535	TK+	666aa	A (5617)
NM_001018065.2	TK–	553aa	B (6081)
NM_001018066	TK–	537aa	B (6081)
NM_001007097	TK–	477aa	C (5123)
NM_001369547	TK–	471aa	C (5123)
NM_001291937.1	TK–	464aa	C (5123)
NM_001369550	TK–	321aa	C (5123)

**Table 2:** Mouse *Ntrk2* mRNA variants

Mouse *Ntrk2* mRNA variants			
ID	Tyrosine kinase (TK) +/–	Coded protein	3′UTR (bp)
NM_001025074.1	TK+	821aa	A (1624)
NM_001282961.1	TK+	821aa	AB (5617)
BC052014	TK–	476aa	C′ (425)
M33385	TK–	476aa	C (1053)
NM_008745.3	TK–	476aa	CD (4967)
XM_006517150.3	TK–	536aa	E (5702)
XM_006517151	TK–	492aa	F (14979)

**Fig. 3:**
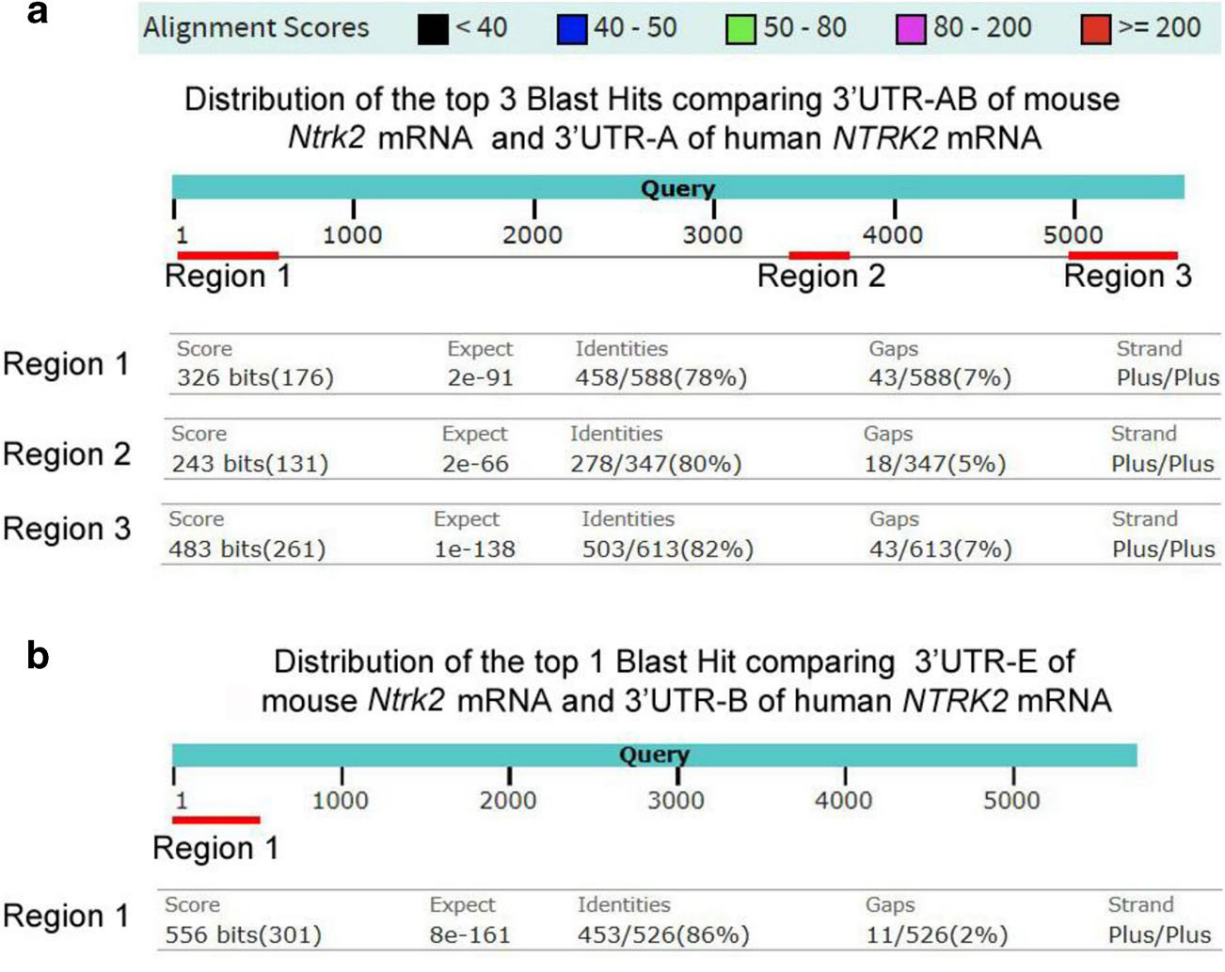
Comparing 3′UTR of *NTRK2* mRNA between mouse and human. Using Basic Local Alignment Search Tool or BLAST to compare the different 3′UTRs of *NTRK2* mRNAs between a mouse and a human. **a** Three regions are shown which got high alignment scores ≥ 200) while comparing 3′UTR-AB of mouse *Ntrk2* mRNA and 3′UTR-A of human *NTRK2* mRNA. Region 1 is located within the first 600-nucleotide region of each 3′UTR or within the 3′UTR-A region of mouse *Ntrk2* mRNA, while the other two regions are located within the 3′UTR-B region of mouse *Ntrk2* mRNA. **b** One region with high alignment scores (≥ 200) is shown while comparing 3′UTR-E of mouse *Ntrk2* mRNA and 3′UTR-B of human *NTRK2* mRNA

To find any conserved sequences in 3′UTRs between human and mouse, we compared 3′UTRs of NTRK2 mRNA between mouse and human using Basic Local Alignment Search Tool or BLAST. There are three homologous regions found from comparing 3′UTR-AB of mouse *Ntrk2* mRNA with 3′UTR-A of human *NTRK2* mRNA (Fig. 3a). Region 1 is located within the 3′UTR-A region of mouse *Ntrk2* mRNA, while the other two regions are located within the 3′UTR-B region of mouse *Ntrk2* mRNA. Only one region with high alignment scores (≥ 200) is found while comparing 3′UTR-E of mouse *Ntrk2* mRNA and 3′UTR-B of human *NTRK2* mRNA (Fig. 3b).

Through different *cis*-regulatory elements present in the sequence, different RNA binding proteins (RBPs) could associate with 3′UTRs to influence transcript stability, localization, and translational efficiency. Based on published top 6-mers for RBPs (Dominguez et al. 2018), potential RBPs and binding sites are shown in the Supplemental Table. There are ten potential RNA binding sites for RBFOX1, HNRNPD, and ZFP36, and four binding sites for DAZAP1, KHSRP, PABPN1L, PUM1, RBMS2/3, and SRSF11 in 3′UTR-B. Truncated NTRK2 3′UTR-D has more than five (including five) potential binding sites for BOLL, CPEB1, ELAVL4, FUBP1, HNRNPC/HNRNPCL1, PUF60, RBFOX1, RBM4, SRSF11, TIA1, and TRNAIUAP. There are at least 3 times more potential RBP binding sites analyzed in our study located in 3′UTR-B and 3′UTR-D than 3′UTR-A and 3′UTR-C (Supplemental Table). Possible reason is that the lengths of 3′UTR-B and 3′UTR-D are longer than 3′UTR-A and 3′UTR-C. Further experiments are needed to confirm RBP roles in regulating *Ntrk2* mRNAs.

### 3′UTRs Regulate Mouse *Ntrk2* mRNA Neuronal Distribution

**Fig. 4:**
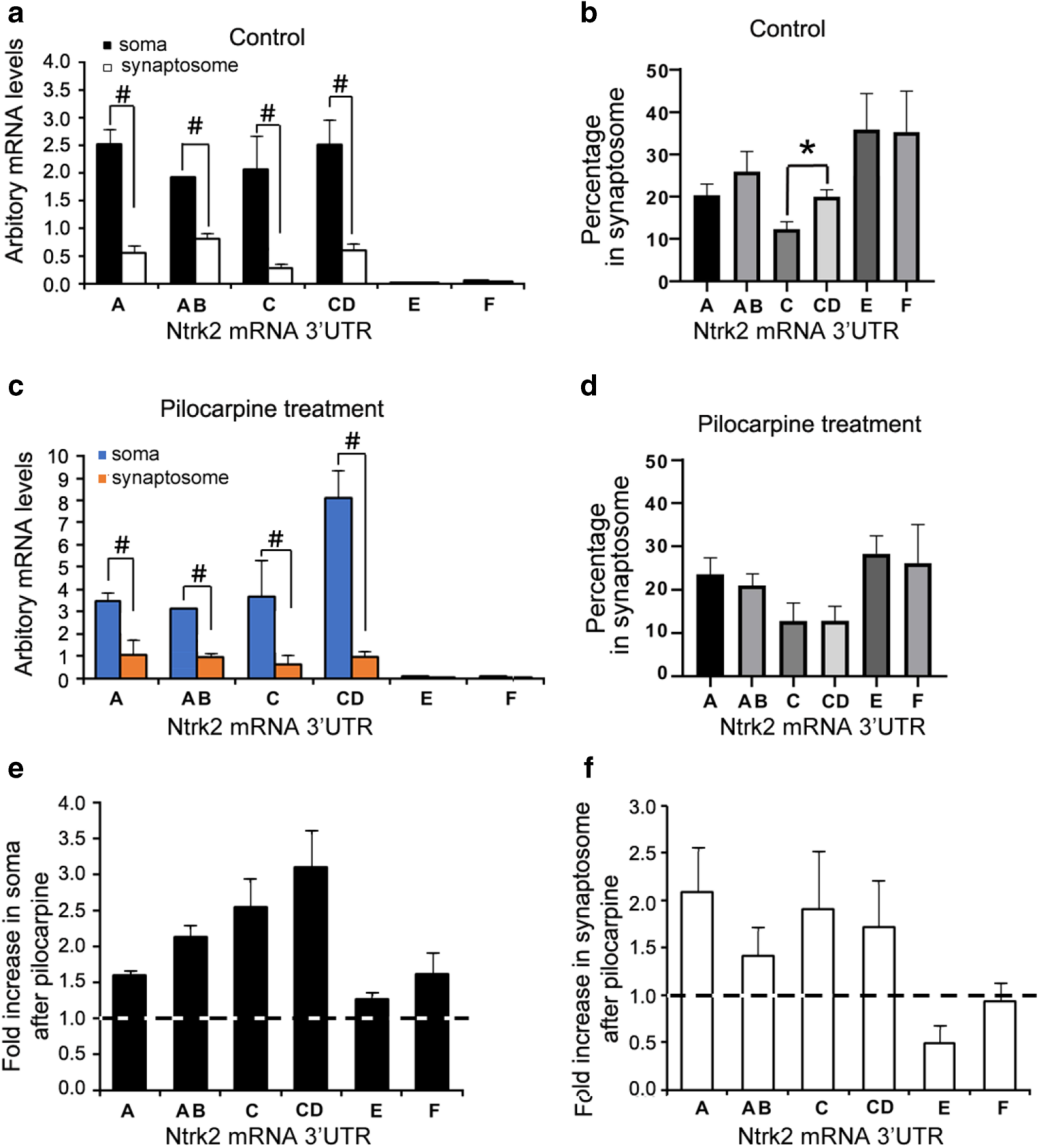
QPCR results showing distribution of mouse *Ntrk2* mRNA isoforms in soma and synaptosome. QPCR was used to analyze the levels of different *Ntrk2* mRNA isoforms in soma and synaptosome, which were isolated from cortex of mice treated with saline (control) or pilocarpine (300 mg/kg). Primer sets A to F were designed to detect different *Ntrk2* mRNA isoforms as demonstrated in Fig. 2. **a** Arbitrary levels of different *Ntrk2* mRNA transcripts in soma or synaptosome calculated from QPCR in controls (*n* = 6). **b** Percentage of each *Ntrk2* mRNA isoform levels in synaptosome from controls. **c** Arbitrary levels of different *Ntrk2* mRNA isoforms in soma or synaptosome after pilocarpine treatment (*n* = 5). **d** Percentage of each *Ntrk2* mRNA isoform levels in synaptosome from animals treated with pilocarpine. **e** Fold changes of different *Ntrk2* mRNA isoforms in soma after pilocarpine treatment compared with those in controls. **f** Fold changes of different *Ntrk2* mRNA isoforms in synaptosome after pilocarpine treatment compared with those in controls. Statistical analysis was performed using an unpaired Student’s *t* test (*, *p* < 0.05; #, *p* < 0.01).

We hypothesize that *Ntrk2* mRNAs with different 3′UTR might affect the cellular distribution of *Ntrk2* mRNA in neurons too. To determine the distribution of mouse *Ntrk2* mRNAs in different areas of neurons, total RNAs were exacted from somata or synaptosomes, which are enriched with elements from distal dendritic regions. Different primer sets are used in QPCR to detect different mouse *Ntrk2* mRNA isoforms (Fig. 2, and Table 3). Primer set A detects *Ntrk2* mRNA with 3′UTR-A (Ntrk2-A) or 3′-UTR AB (Ntrk2-AB), primer set B detects Ntrk2-AB, primer set C detects *Ntrk2* mRNA with 3′UTR-C (Ntrk2-C) or 3′-UTR CD (Ntrk2-CD), primer set D detects Ntrk2-CD, primer set E detects *Ntrk2* mRNA with 3′UTR-E (Ntrk2-E), and primer set F detects *Ntrk2* mRNA with 3′UTR-F (Ntrk2-F). Meanwhile, a previous study showed that pro-epileptic drug pilocarpine can induce mRNA transportation into dendrites (Vicario et al. 2015); we used pilocarpine as stimulation to study whether pilocarpine could influence the levels or distribution of *Ntrk2* mRNAs in neurons. With or without pilocarpine stimulation, levels of all detected *Ntrk2* mRNA isoforms in somata were significantly higher than those in synaptosomes (Fig. 4a, c). Based on our QPCR results, 27%, 25%, 20%, 27%, 0.3%, and 0.7% of the total *Ntrk2* transcripts detected are Ntrk2-A, Ntrk2-AB, Ntrk2-C, Ntrk2-CD, Ntrk2-E, and Ntrk2-F, respectively (calculated from data shown in Fig. 4a). We did not add QPCR results for Ntrk2 mRNA with 3′UTR-C′ because of very low levels (data not shown). Within Ntrk2-AB, 25% were located in synaptosomes, slightly higher (not significant) than Ntrk2-A and Ntrk2-AB combined in synaptosomes (20%) (Fig. 4b). A total of 20% Ntrk2-CD were located in synaptosomes, significantly higher (*p* < 0.05) than Ntrk2-C and Ntrk2-CD combined levels in synaptosomes (10%) (Fig. 4b). After pilocarpine treatment, all *Ntrk2* mRNA transcripts in somata were increased compared with that in controls (Fig. 4e), while the increases in synaptosomes were smaller (Fig. 4f). Therefore, the proportions of *Ntrk2* transcripts in synaptosomes were all smaller after pilocarpine treatment (Fig. 4d). A total of 21%, 18%, 19%, 41%, 0.2%, and 0.8% of tested *Ntrk2* transcripts are Ntrk2-A, Ntrk2-AB, Ntrk2-C, Ntrk2-CD, Ntrk2-E, and Ntrk2-F, respectively, after pilocarpine treatment (calculated based on data shown in Fig. 4c).

**Table 3:** The sequences of primers used in cloning or RT-PCR

Purpose	Target	Forward primer	Reverse primer
Cloning	3′UTR-A of *Ntrk2* mRNA	GGGTCCTCCTTCTGCCCAGACCGTCCTTCC	TAACTTTTTAATGGAGTATAGTTAACAA
Cloning	3′UTR-B of *Ntrk2* mRNA	GAATTAAGCCTTGACACTGTATGGCTGACA	ACCTCTTGTGGAAGATGTTATTTATTG
Cloning	3′UTR-AB of *Ntrk2* mRNA	GGGTCCTCCTTCTGCCCAGACCGTCCTTCC	ACCTCTTGTGGAAGATGTTATTTATTG
RT-PCR	β-actin	AAGTCCCTCACCCTCCCAAAAG	AAGCAATGCTGTCACCTTCCC
Primer set A RT-PCR	*Ntrk2* mRNA with 3′UTR-A or 3′-UTR AB	TGACCAATCTGGCTTCTGCATTCC	GGTGGGCAAACTGGAGTGTCTG
Primer set B RT-PCR	*Ntrk2* mRNA with 3′UTR AB	GGTACTGTCAGGCACTGTGGAAC	TAGGTCACGGCTGGCGGAAG
Primer set C RT-PCR	*Ntrk2* mRNA with 3′UTR C or with 3′UTR CD	GCAGGTAGAACGGAGCAGCAC	CAGAGGGCAATGGAAAGGGACAAG
Primer set D RT-PCR	*Ntrk2* mRNA with 3′UTR D	GGACCGCCATCAGCAACACAG	TCCCTTCTCTCTTCCTGGCACTG
Primer se E RT-PCR	*Ntrk2* mRNA with 3′UTR-E	CTGACAGCATGGGGTGGTTGAC	GGCAAGGCAAGGAGAAGATAGCAC
Primer set F RT-PCR	*Ntrk2* mRNA with 3′UTR-F	CAAGACTTGGCGGTGACCTGTG	AGGGAGGCAACATCAGAAAAGACC

**Fig. 5:**
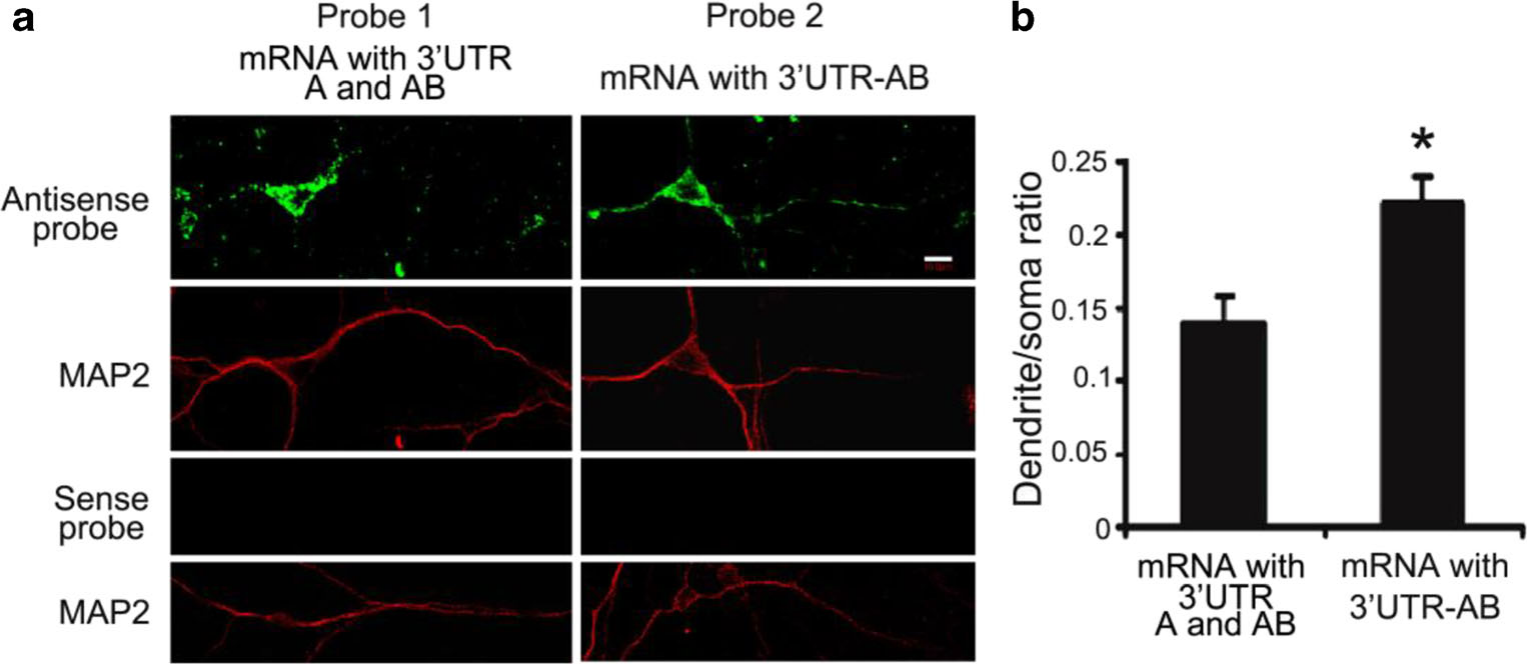
mRNA encoding full-length *Ntrk2* with long 3′UTR-AB is distributed more in dendrites. **a** Riboprobes were derived from the coding region (probe 1) or 3′UTR-AB (probe 2) of mRNA encoding mouse full-length *Ntrk2* (Fig. 2). Representative images from FISH show the distribution of full-length *Ntrk2* mRNA in cell bodies and dendrites of cultured rat hippocampal neurons. MAP2 immunostaining marks cell bodies and dendrites of neurons. The scale bar represents 10 μm. **b** Ratio of FISH signals in dendrites to those in cell bodies. Data were obtained from 30 transfected neurons from three independent experiments. Error bars indicate standard errors. Data were analyzed with an unpaired Student’s *t* test (*, *p* < 0.05)

**Fig. 6:**
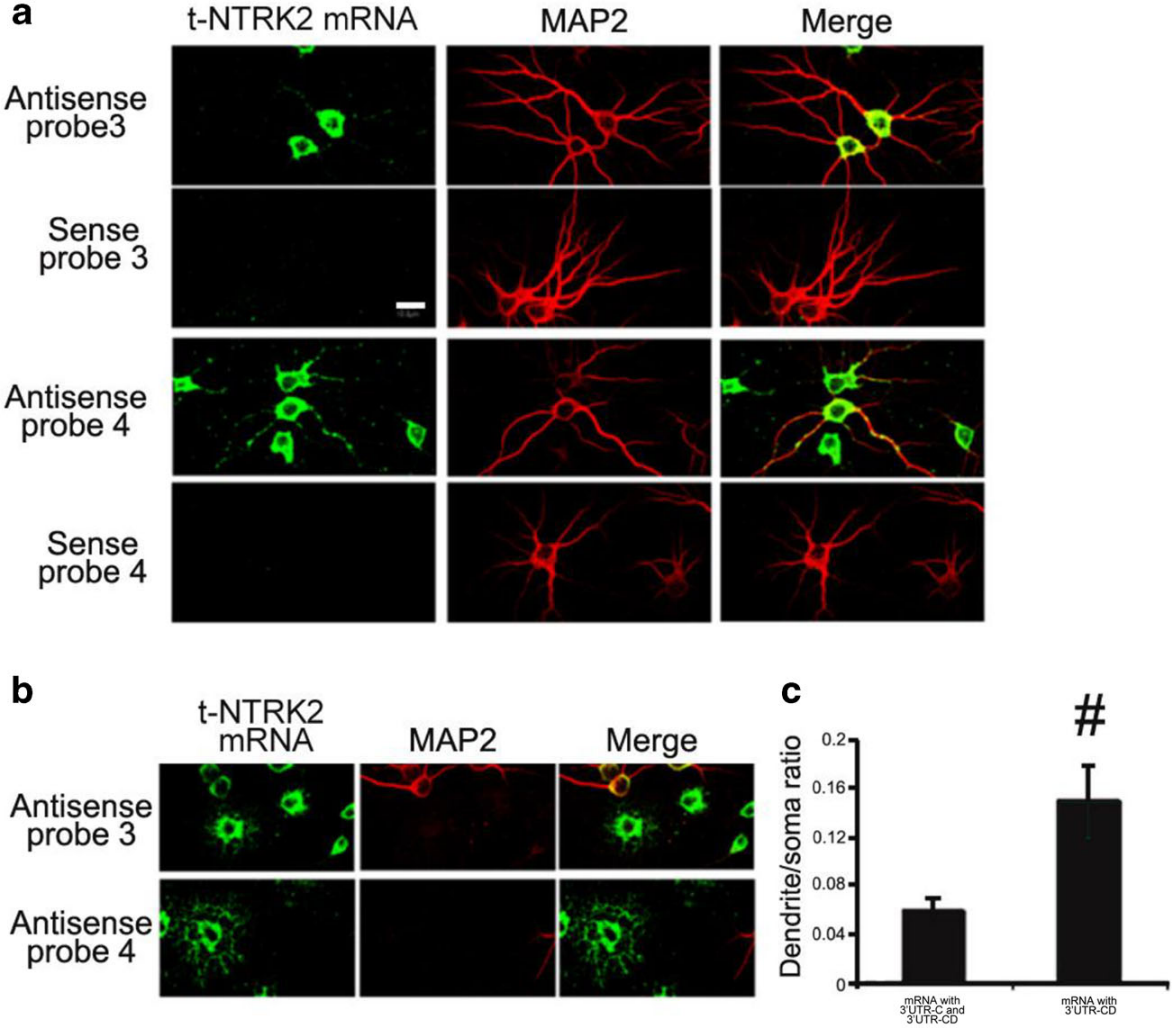
mRNA encoding truncated mouse *Ntrk2* with long 3′UTR-CD is distributed more in dendrites. **a** Riboprobes were derived from the coding region (probe 3) or 3′UTR-CD (probe 4) of mRNA encoding truncated *Ntrk2* (Fig. 2). Representative images from FISH show the distribution of truncated *Ntrk2* mRNA in cell bodies and dendrites of cultured rat cortical neurons. MAP2 immunostaining marks cell bodies and dendrites. The scale bar represents 20 μm. **b** Representative images from FISH also show truncated *Ntrk2* mRNA with 3′UTR-CD is distributed more in extended process of glial cells. **c** Ratio of FISH signals in dendrites to those in cell bodies of cultured neurons. Error bars indicate standard errors. Data were obtained from more than 30 transfected neurons from three independent experiments and analyzed with an unpaired Student’s *t* test (#, *p* < 0.01)

To directly observe the distribution of different mouse *Ntrk2* mRNA variants encoding full-length NTRK2 in neurons, we used highly sensitive fluorescence in situ hybridization (FISH) on cultured mouse cortical neurons. Two sets of RNA probes are generated to detect both Ntrk2-A and Ntrk2-AB (probe 1) or Ntrk2-AB only (probe 2), respectively (Fig. 2). It is obvious that majority of mRNA variants are localized in soma, especially results from probe 1, which recognizes both Ntrk2-A and Ntrk2-AB (Fig. 5a first column). Similarly, FISH signals were mainly detected in soma and proximal dendrites transfected with GFP-bovine growth hormone (BGH) construct using previously described BGH specific probe (An et al. 2008) (data not shown). However, FISH signal in dendrites was stronger using probe 2, which only recognized Ntrk2-AB (Fig. 5a second column). Quantification of FISH signals demonstrated that the ratio of the FISH signal in the initial 50-μm segment of dendrites to the somatic FISH signals from probe 2 was significantly higher than those from probe 1 (Fig. 5b). Similar FISH signals in distal dendrites were detected using positive probes recognizing long 3′UTR of BDNF mRNA (data not shown), consistent with a previous report (An et al. 2008). Therefore, more Ntrk2-AB is targeted to dendrites than Ntrk2-A. We then studied the distribution of truncated *Ntrk2* (*t-NTRK2*) mRNAs with different 3′UTR (Ntrk2-C and Ntrk2-CD). Two sets of RNA probes were generated to detect both Ntrk2-C and Ntrk2-CD (probe 3) or only Ntrk2-CD (probe 4), respectively. Results from FISH demonstrated that more Ntrk2-CD transcripts were distributed to dendrites compared with Ntrk2-C and Ntrk2-CD combined (Fig. 6). In addition of its expression in neurons, t-NTRK2 was also expressed in glial cells (Fig. 6b), consistent with other’s report (Tushev et al. 2018). Interestingly, more Ntrk2-CD transcripts were located in distal processes than Ntrk2-C and Ntrk2-D combined (Fig. 6b).

**Fig. 7:**
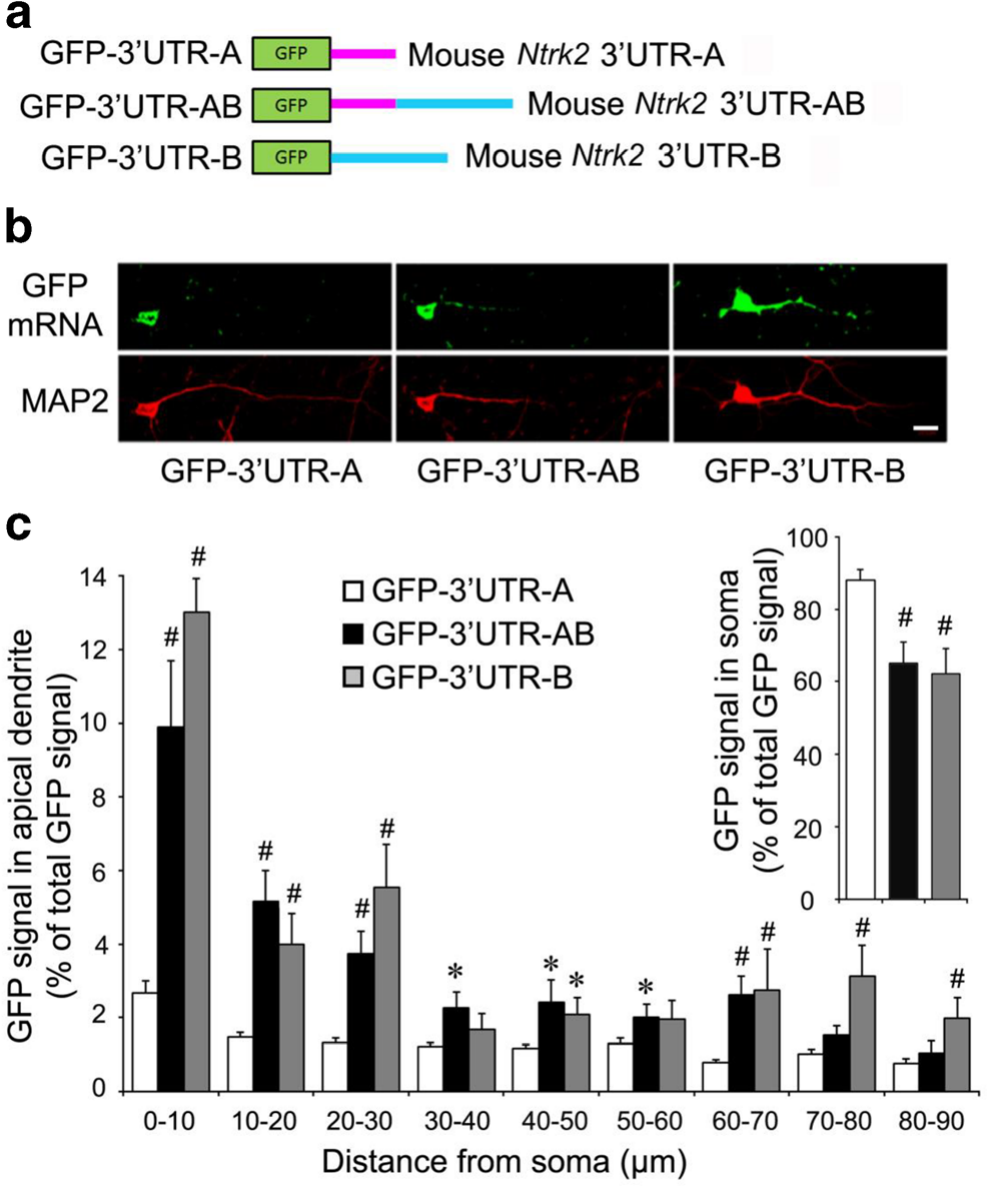
*cis*-Acting sequence in the 3′UTR is required for the dendritic localization. **a** Schematic of three GFP constructs with different mouse *Ntrk2* 3′UTRs. **b** Localization of GFP mRNA in neurons transfected with different GFP constructs. Top panels: FISH of cultured neurons with a GFP antisense riboprobe. Bottom panels: MAP2 immunocytochemistry. The scale bar represents 20 μm. **c** Percentage of GFP signals in soma (insert on right) or apical dendrites of total GFP signals (combination of GFP signals in soma and apical dendrite). GFP signals in apical dendrites from GFP-3′UTR-A expression were compared with those from the GFP-3′UTR-B or GFP-3′UTR-AB. Error bars indicate standard errors. Data were obtained from > 30 transfected neurons with each construct from three independent experiments and analyzed with an unpaired Student’s *t* test (*, *p* < 0.05; #, *p* < 0.01)

Because the primers or probes used in our QPCR or FISH experiments to detect endogenous Ntrk2 mRNA levels could not distinguish Ntrk2-AB from Ntrk2-A or Ntrk2-CD from Nrk2-C, Ntrk2-A and Ntrk2-C levels could not be detected directly. Therefore, to further confirm 3′UTR of mouse *Ntrk2* mRNA effects on cellular distribution of mRNA, we developed three constructs generated by fusion of the cDNA for green fluorescence protein (GFP) to a sequence encoding the 3′UTR-A, 3′UTR-AB, or 3′UTR-B of mouse NTRK2 mRNAs (Fig. 7a). Expressed fusion transcripts in cultured mouse cortical neurons were examined via FISH with antisense probes corresponding to the GFP coding sequence. Percentage of FISH signals in somata or apical dendrites of total FISH signals (combination of FISH signals in soma and apical dendrite) was calculated (Fig. 7c). FISH signals from GFP-3′UTR-A expression were compared with those from the expression of the other two constructs. Majority of FISH signals were detected in soma (> 80% of total FISH signals) and proximal dendrites of neurons transfected with the GFP-3′UTR-A construct (Fig. 7b, c). In contrast, more FISH signals were detected in distal dendrites in neurons transfected with either the GFP-3′UTR-AB or GFP-3′UTR-B construct (Fig. 7 b and c). Levels of GFP mRNA at any 10 μm dendritic segment were significantly higher in neurons expressing GFP-3′UTR-AB or GFP-3′UTR-B than in neurons expressing GFP-3′UTR-A (Fig. 7c). These results are consistent with our observations in Fig. 6. Taken together, these results showed that the *Ntrk2* 3′UTR sequence was sufficient to target GFP mRNA to dendrites.

## Discussion

In humans, BDNF mutations contribute to neurodegenerative disorders such as Alzheimer’s, Huntington’s, and Parkinson diseases (Conover et al. 1995; Murer et al. 2001; Siegel and Chauhan 2000; Zuccato and Cattaneo 2007). As BDNF receptor, full-length NTRK2 mediates the effects of BDNF through PLCγ/PI3K/MAPK pathways (Huang and Reichardt 2001; Numakawa et al. 2010). Manipulations of the levels or activity of NTRK2 result in similar or more severe effects compared with BDNF malfunctions, implying that NTRK2 has broader effects than BDNF itself (reviewed in (Minichiello 2009)).

As demonstrated in Figs. 1 and 2, NTRK2 protein is structurally very similar between a human and a mouse. In mouse, there are one NTRK2 TK(+) (821aa) and three NTRK2 TK(−) isoforms: 476aa isoform with unique short C-terminal amino acid sequence of 11 aa (also called TrkB.T1), 492aa isoform with unique short C-terminal amino acid sequence of 27 aa, and 536aa isoform. In humans, there exist more isoforms of NTRK2: TK(+) isoforms include 838aa, 822aa, 810aa, 682aa, and 666aa; TK(−) isoforms include 553aa/537aa with unique short C-terminal amino acid sequence of 9 aa, 477aa/464aa/321aa with unique short C-terminal amino acid sequence of 12 aa, and 471aa with unique short C-terminal amino acid sequence of 6 aa. Based on our data and other’s report, NTRK2 TK(+) and TrkB.T1 transcripts combined account for 95% of total *Ntrk2* mRNA (Klein et al. 1989; Luberg et al. 2010; Stoilov et al. 2002; Tomassoni-Ardori et al. 2019). Therefore, it is not surprising that majority of studies on NTRK2 focused on TK(+) and TrkB.T1. The intracellular domains of NTRK2 TK(−) isoforms exhibit 100% sequence conservation between a human and a mouse. 821aa mouse NTRK2 isoform is 93% similar to 822aa human NTRK2 isoform (data not shown). Because of these similarity between human and mouse NTRK2, suggesting the functions of NTRK2 in human and mouse are similar too. TrkB.T1 plays a dominant negative role to inhibit the activation of NTRK2 TK(+) through forming a heterodimer with NTRK2 TK(+) (Eide et al. 1996; Haapasalo et al. 2001; Li et al. 1998). In addition, TrkB.T1 can form a homodimer to regulate cytoskeletal changes in a BDNF-independent manner in both neurons and glial cells (Cheng et al. 2007; Ohira et al. 2006; Yacoubian and Lo 2000). TrkB.T1 can also activate distinct intracellular signaling pathways different from NTRK2 TK(+) (Cheng et al. 2007; Ohira et al. 2005; Rose et al. 2003). The functions of other NTRK2 TK(−) isoforms are still largely unknown.

In addition to the similarity in NTRK2 protein structure, our analysis showed several homologous regions in 3′UTRs of NTRK2 mRNA between a human and a mouse. There are three conserved regions in the 3′UTR-A of human *NTRK2* mRNA or 3′UTR-AB of mouse *Ntrk2* mRNA, one conserved region in 3′UTR-B of human *NTRK2* mRNA and 3′UTR-E of mouse *Ntrk2* mRNA. Because of these conserved structures and sequences between mouse and human NTRK2, mouse models are useful tools to study the physiology of human NTRK2.

Both NTRK2 TK(+) and TrkB.T1 proteins are widely expressed in the central neuron system (reviewed in (Fenner 2012). In this study, we examined different mouse *Ntrk2* mRNA variant expression profiles in soma or synaptosome of mouse cortex with or without pilocarpine stimulation. Pilocarpine increased the levels of all *Ntrk2* mRNA transcripts in somata; however, the increases in synaptosomes were smaller. It was reported that *Ntrk2* mRNAs with long 3′UTRs encoding full-length or truncated NTRK2 are the mostly expressed isoforms, accounting together for 95% of the total *Ntrk2* transcripts (Tomassoni-Ardori et al. 2019). Based on our QPCR results, *Ntrk2* mRNA with long 3′UTR-AB or 3′UTR-CD combined accounts for 53% of the total *Ntrk2* transcripts. *Ntrk2* mRNA with short 3′UTR-A or B combined account for about 47%. The possible discrepancies are that we analyzed mouse cortex while they analyzed mouse hippocampi and employed different methods. The percentage of *Ntrk2* mRNA transcripts encoding full-length NTRK2 (Ntrk2-A and Ntrk2-AB combined) is similar to those encoding NTRK2 TK(−) (Ntrk2-C, Ntrk2-CD, Ntrk2-E, and Ntrk2-F combined).

QPCR results also showed that small portions of all *Ntrk2* mRNA variants were detected in synaptosomes. Significant differences were observed in synaptosome distributions between *Ntrk2* mRNA with 3′UTR-C and 3′UTR-CD, but not between 3′UTR-A and 3′UTR-AB. However, FISH results showed increased levels of *Ntrk2* mRNA with long 3′UTR-AB or CD in apical dendrites compared with mRNA with short 3′UTR-B or C, respectively. RNAs used in QPCR were extracted from somata or synaptosomes of mouse cortex. Although synaptosomes isolated from brain tissues are enriched with elements from distal dendritic regions, they also contain other elements. Therefore, synaptosomes might not be as accurate as FISH signals visually showing mRNA distribution in the dendrites of cultured neurons. After pilocarpine treatment, all mRNA levels of Ntrk2-A, Ntrk2-AB, Ntrk2-C, or Ntrk2-CD in soma were increased compared with those of controls, while the increases in synaptosomes were small. Then, the proportions of *Ntrk2* transcripts in synaptosomes were all smaller after pilocarpine treatment, which resulted in no significant differences in dendritic mRNA levels between 3′UTR-A and 3′UTR-AB, or 3′UTR-C and 3′UTR-CD after pilocarpine treatment. Our data also showed that *Ntrk2* mRNA encoding for TrkB.T1 was expressed in glial cells. This is consistent with previous reports (Ohira et al. 2005; Rose et al. 2003; Tushev et al. 2018).

Studies have shown that nervous system specifically has a broad range of transcripts with different 3′UTRs (Ciolli Mattioli et al. 2019). Many of these 3′UTRs help mRNA dendritic localization, allowing neurons to regulate local translation in prompt response to local stimulation or in urgent need. However, the status and mechanisms of subcellular compartmentalization of different mRNA isoforms of a specific gene are controversial. Previous studies indicate that BDNF transcripts encoding same BDNF protein also have two different 3′UTRs, with either a short or a long 3′UTR. The short 3′UTR BDNF mRNAs are restricted to somata, whereas the long 3′UTR mRNAs are also localized in dendrites and responsible for the dendritic targeting of BDNF mRNAs (An et al. 2008). However, later reports indicated that *BDNF* mRNAs with either 3′UTRs could be transported in dendrites in response to neural activity (Baj et al. 2011; Oe and Yoneda 2010; Vicario et al. 2015). Despite these discrepancies, it is clear that different 3′UTRs of one gene are involved in selective targeting mRNA in response to different stimuli through associating with different sets of RNA binding proteins (Vicario et al. 2015), and the regulations of gene expression at mRNA levels are more complicated than originally hypothesized.

The dendritic localization of mRNA might result from several mechanisms that can work independently or in a coordinated way: (a) Motor proteins directly help the transport of mRNAs; (b) Associated with pre-localized anchoring proteins, mRNAs diffuse into distal destinations; (c) Trans-acting factors bind to specific *cis*-regulatory elements present in a mRNA and regulate the distribution of this mRNA (Medioni et al. 2012). Different 3′UTRs suggest different microRNA sites or different *cis*-regulatory elements for RNA binding proteins, which might influence transcript stability, localization, and translational efficiency. Structurally, NTRK2 proteins are very similar between a human and a mouse. Furthermore, several conserved regions exist in the 3′UTRs between human and mouse Ntrk2 mRNAs. These homologous regions and associated RBPs might play important roles in regulating the expression and distribution of both human and mouse NTRK2 mRNA. Our supplemental table provides potential RBPs for *Ntrk2* mRNAs based on consensus-binding motifs. For example, motif (U)GCAUG, the binding site for RNA binding protein RBFOX1, was found in *Ntrk2* 3′UTR-A, AB, and CD. Indeed, RBFOX1 is the first RBP shown to regulate *Ntrk2* expression at the mRNA level (Tomassoni-Ardori et al. 2019). The upregulation of RBFOX1 selectively increases hippocampal truncated Ntrk2 isoform expression (Tomassoni-Ardori et al. 2019). It is worthy in future studies to identify RNA binding proteins on *Ntrk2* mRNA processing.

## Electronic Supplementary Material

ESM 1 (DOCX 35 kb)
