# Supplementary material for: 3′UTRs Regulate Mouse Ntrk2 mRNA Distribution in Cortical Neurons
Source: J Mol Neurosci. 2020 May 19;70(11):1858–70. doi: 10.1007/s12031-020-01579-8 (PMC7561570; doi:10.1007/s12031-020-01579-8)
Supplement: Supplementary file 1 — (DOCX 35 kb) [file 12031_2020_1579_MOESM1_ESM.docx]

**Supplementary Table: potential cis-acting elements for RNA binding proteins (RBP) on mouse *Ntrk2* mRNA 3’UTRs.**

| RBP | RBP binding  (best contiguous  6-mers) | Full length *Ntrk2* 3’UTR | | Truncated *Ntrk2* 3’UTR | |
| --- | --- | --- | --- | --- | --- |
|  |  | A | B | C | D |
| A1CF | UAAUUA | 0 | 3 | 0 | 1 |
| BOLL | UUUUUU | 0 | 1 | 0 | 5 |
| CELF1 | GUUUGU | 1 | 3 | 1 | 0 |
| CNOT4 | CACACA | 0 | 2 | 1 | 1 |
| CPEB1 | UUUUUA | 0 | 3 | 0 | 8 |
| DAZ3 | AGUUUA | 0 | 0 | 0 | 2 |
| DAZAP1 | UAUAUA | 0 | 4 | 0 | 0 |
| EIF4G2 | GUUGCA | 0 | 2 | 0 | 0 |
| ELAVL4 | UUUUUU | 0 | 1 | 0 | 5 |
| ESRP1 | GGGGGG | 0 | 0 | 0 | 1 |
| EWSR1 | GGGGGG | 0 | 0 | 0 | 1 |
| FUBP1 | UUUUUU | 0 | 1 | 0 | 5 |
| FUBP3 | AUAUAU | 0 | 3 | 0 | 1 |
| HNRNPC | UUUUUU | 0 | 1 | 0 | 5 |
| HNRNPCL1 | UUUUUU | 0 | 1 | 0 | 5 |
| HNRNPD | UAUUUA | 6 | 10 | 1 | 0 |
| HNRNPDL | UAAUUA | 0 | 3 | 0 | 1 |
| HNPNPK | CAGCCC | 1 | 3 | 0 | 0 |
| HNRNPL | CACACA | 0 | 2 | 1 | 1 |
| IGF2BP1 | ACAAUA | 0 | 2 | 1 | 1 |
| IGF2BP2 | CACACA | 0 | 2 | 1 | 1 |
| KHDRBS2 | AAAUAA | 0 | 3 | 1 | 1 |
| KHDRBS3 | AAUAAA | 0 | 3 | 1 | 2 |
| KHSRP | AUGUAU | 0 | 4 | 0 | 0 |
| MBNL1 | GCUUGC | 1 | 0 | 0 | 0 |
| NOVA1 | AUUCAU | 1 | 3 | 0 | 2 |
| PABPN1L | AAAAAA | 2 | 7 | 0 | 4 |
| PCBP1 | CCGCCC | 1 | 0 | 0 | 0 |
| PCBP2 | CCCCCC | 1 | 0 | 0 | 2 |
| PCBP4 | AUCCCC | 0 | 1 | 0 | 0 |
| PRR3 | AUAAGC | 0 | 1 | 0 | 0 |
| PTBP3 | CUUUCU | 3 | 2 | 0 | 3 |
| PUF60 | UUUUUU | 0 | 1 | 0 | 5 |
| PUM1 | UGUAUA | 0 | 4 | 0 | 0 |
| RALYL | UUUUUU | 0 | 1 | 0 | 5 |
| RBFOX1 | (U)GCAUG | 4 | 1 | 0 | 6 |
| RBFOX3 | GCAUGC | 1 | 1 | 0 | 2 |
| RBM4 | UUUUUU | 0 | 1 | 0 | 5 |
| RBM4B | UACCGG | 0 | 1 | 0 | 0 |
| RBM6 | CCCCCC | 1 | 0 | 0 | 2 |
| RBM15B | GUGUGU | 1 | 2 | 0 | 1 |
| RBM22 | GGGGGG | 0 | 0 | 0 | 1 |
| RBM24 | UACUUU | 0 | 1 | 0 | 1 |
| RBM25 | ACUUAC | 0 | 0 | 0 | 1 |
| RBM47 | CGUCCC | 0 | 1 | 0 | 1 |
| RBMS2 | UAUAUA | 0 | 4 | 0 | 0 |
| RBMS3 | UAUAUA | 0 | 4 | 0 | 0 |
| SF1 | UAACAA | 2 | 0 | 0 | 3 |
| SFPQ | UGUAAG | 0 | 0 | 0 | 1 |
| SNRPA | UGCACA | 0 | 2 | 1 | 2 |
| SRSF2 | AGCAGC | 1 | 1 | 2 | 3 |
| SRSF4 | AAGCAG | 0 | 2 | 1 | 1 |
| SRSF5 | AGCAGC | 1 | 1 | 2 | 3 |
| SRSF8 | AGCAGU | 0 | 2 | 0 | 2 |
| SRSF9 | GCAGCC | 1 | 3 | 1 | 1 |
| SRSF10 | AGCAGC | 1 | 1 | 2 | 3 |
| SRSF11 | AGGAGA | 3 | 5 | 0 | 5 |
| TAF15 | GGGGGG | 0 | 0 | 0 | 1 |
| TARDBP | AGUAUG | 0 | 1 | 1 | 1 |
| TIA1 | UUUUUU | 0 | 1 | 0 | 5 |
| TRA2A | GAAGAA | 0 | 1 | 1 | 1 |
| TRNA1UAP | UUUUUU | 0 | 1 | 0 | 5 |
| UNK | AAUUAG | 0 | 0 | 0 | 1 |
| ZCRB1 | AUUAAU | 1 | 1 | 1 | 0 |
| ZFP36 | UAUUUA | 6 | 10 | 1 | 0 |
| ZFP36 | UAUUUA | 6 | 10 | 1 | 0 |
| Total analyzed RBP binding sites | | 37 | 124 | 21 | 120 |
